# A novel preventive strategy against HIV-1 infection: combinatorial use of inhibitors targeting the nucleocapsid and fusion proteins

**DOI:** 10.1038/emi.2017.26

**Published:** 2017-06-07

**Authors:** Yu Yang, Jingyu Zhu, Matthew Hassink, Lisa M Miller Jenkins, Yanmin Wan, Daniel H Appella, Jianqing Xu, Ettore Appella, Xiaoyan Zhang

**Affiliations:** 1 Scientific Research Center, Shanghai Public Health Clinical Center and Institutes of Biomedical Sciences, Key Laboratory of Medical Molecular Virology, Fudan University, Shanghai 201508, China; 2 Synthetic Bioactive Molecules Section, Laboratory of Bioorganic Chemistry, National Institute of Diabetes and Digestive and Kidney Diseases, NIH, Bethesda, MD 20814, USA; 3 Chemical Immunology Section, Laboratory of Cell Biology, Center for Cancer Research, National Cancer Institute, NIH, Bethesda, MD 20892, USA

**Keywords:** combinatorial therapy, HIV-1, inhibitor, viral transmission

## Abstract

The strategy of simultaneously attacking multiple targets is worthy of exploration in the field of microbicide development to combat HIV-1 sequence diversity and minimize the transmission of resistant variants. A combination of *S*-acyl-2-mercaptobenzamide thioester-10 (SAMT10), an inhibitor of the HIV-1 nucleocapsid protein (NCp7), and the fusion inhibitor sifuvirtide (SFT) may exert synergistic effects, since SFT can block viral fusion at an early stage of the viral cycle and SAMT10 can disrupt viral particles at a later stage. In this study, we investigated the effect of the combination of SAMT10 and SFT on HIV-1 infection using *in vitro* cell culture and *ex vivo* mucosal explant models. A range of doses for each compound was tested at 10-fold serial dilutions based on their 50% effective concentrations (EC_50_). We observed a synergistic effect of SAMT10 and SFT *in vitro* against both the laboratory-adapted HIV-1 strain HIV-1_IIIB_ (subtype B, X4) and three pseudotyped viruses prevalent in Chinese sexually transmitted populations (SVPB16 (subtype B, R5), SVPC12 (subtype C, R5) and SH1.81 (CRF01_AE, R5)). In the *ex vivo* study, the EC_50_ values of the inhibitor combinations were reduced 1.5- to 2-fold in colorectal mucosal explants compared to treatment with SAMT10 or SFT alone by using with HIV-1_IIIB_. These results may provide a novel strategy for microbicide development against HIV-1 sexual transmission.

## INTRODUCTION

In 2015, an estimated 1.9 million (1.7–2.2 million) adults were newly infected with HIV-1 globally, and the vast majority acquired HIV-1 through sexual transmission.^1^ Due to the lack of effective vaccines, alternative biomedical prevention strategies are urgently needed to specifically protect people from HIV-1 infection. One strategy being explored is the use of antiviral drugs, such as microbicides. A clinical trial (CAPRISA 004) conducted in South Africa using 1% tenofovir, a reverse transcriptase inhibitor, gel within 12 h before and after sex showed moderate protection against HIV-1 heterosexual transmission.^2^ Although the vaginal formulation of tenofovir was not optimal for rectal application, a reduced glycerin vaginal formulation was found to be better tolerated in the MTN-007 study and is being evaluated in a phase 2 expanded-safety study (MTN-017).^3^ A rectal-specific formulation of tenofovir was also found to be safe and efficacious in preclinical evaluations.^4^ Similar to the field of vaccine and antiretroviral therapy (ART) drug development, the genetic diversity of HIV also poses a great challenge to the development of microbicides. The strategy of simultaneously attacking multiple targets is worthy of exploration in the field of microbicide development to effectively block HIV-1 sexual transmission. Studies using combinations of entry inhibitors with nucleotide, nucleoside and non-nucleoside reverse transcriptase inhibitors in explants and nonhuman primates demonstrated the potential efficacy of rectal microbicides to prevent HIV-1 transmission.^5, 6, 7, 8^ The combination of CMPD167, a CCR5 inhibitor, with BMS-378806 that binds gp120 and C52L, a gp41 binder, resulted in the synergistic inhibition of simian–human immunodeficiency virus (SHIV) infection of macaques.^9^ The Combination of HIV-1 Antiretroviral Rectal Microbicide (CHARM)-01 and CHARM-02 studies further demonstrated the safety, acceptability, luminal distribution and clearance of the reduced glycerin and rectal formations.^10, 11^ These results have resulted in the development of new and more effective microbicides, in which combinations of antiviral molecules exert synergistic effects to provide better protection against HIV-1 infection.

**Figure 1:**
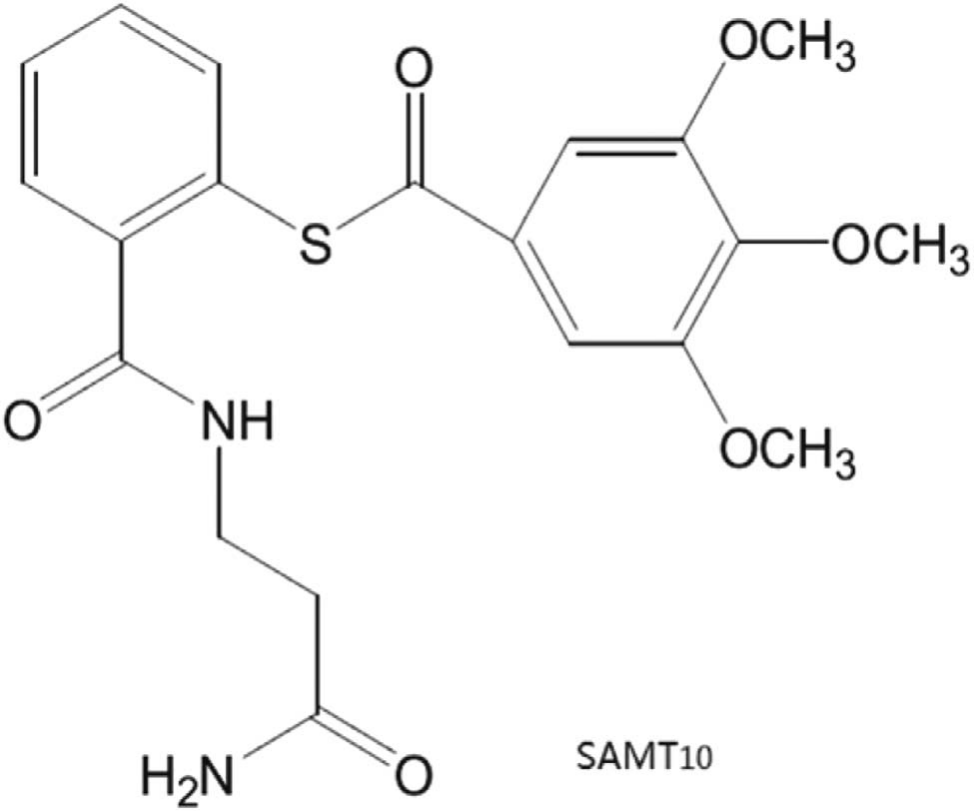
Chemical structure of SAMT10. *S*-acyl-2-mercaptobenzamide thioester-10, SAMT10.

We and other groups found that *S*-acyl-2-mercaptobenzamide thioester compounds (SAMTs), which are HIV-1 nucleocapsid (NCp7) inhibitors that target the second Zn^2+^-finger (ZF2) in the highly conserved NCp7 region (Figure 1), promote zinc ejection from ZF2 and cause cross-linking of NCp7 that prevents Gag processing and results in the production of noninfectious viral particles.^12, 13, 14^ SAMTs are efficacious in *ex vivo* cervico-vaginal explants, transgenic mouse models and rhesus macaques following vaginal challenge.^15^ A new generation of the fusion inhibitor, sifuvirtide (SFT), comprising 36 amino-acid residues that share some sequence and structural features with the native C-terminal heptad repeat peptide, is active against diverse primary and laboratory-adapted HIV-1 strains, and it also shows activity against viruses resistant to the first generation of the fusion inhibitor enfuvirtide.^16, 17, 18^ Our previous studies showed that SFT was well tolerated when administered in a gel formulation in the vaginal cavity of mice and by subcutaneous injection in a phase Ia clinical study.^16^ Furthermore, SFT was efficacious against simian immunodeficiency virus intra-rectal challenge in nonhuman primates.^19^ Therefore, the combination of SAMTs and SFT may exert synergistic effects because SFT can block viral fusion at an early stage of the viral cycle and because SAMTs can disrupt viral particles at a later stage and target HIV-1 reverse transcriptase and Tat during the early phase of the HIV-1 replication cycle.^12^

In this study, we investigated the *in vitro* and *ex vivo* safety and efficacy of SAMT10 in combination with SFT in cultured human colorectal mucosal explants and cellular models. We demonstrated that the combination of SAMT10 and SFT was synergistic and inhibited HIV-1 transmission in preclinical models of HIV replication.

## MATERIALS AND METHODS

### Compounds and reagents

Sifuvirtide was provided by FusoGen Pharmaceuticals, Inc. (Tianjin, China). SAMT10 was synthesized as previously described.^20^ A 3-(4,5-dimethyl-2-thiazyl)-2,5-diphenyl-2H-tetrazolium bromide (MTT) powder was purchased from Thermo Fisher Scientific (Waltham, MA, USA), and nonoxynol-9 (N-9) was purchased from ScienceLab.com, Inc. (Houston, TX, USA). For *in vitro* cell experiments, MTT, N-9, and SFT were dissolved in sterile phosphate-buffered saline (PBS, pH=7.4) at the indicated concentrations. SAMT10 was initially dissolved in a 100 mM stock of dimethyl sulfoxide (DMSO; Sigma-Aldrich, St Louis, MO, USA) and then serially diluted in complete medium for use in experiments. The highest concentration of DMSO used in the cultures did not exceed 0.1%.

### Cell lines and culture

The human T-cell leukemia cell line MT4 was purchased from the Cell Bank of Type Culture Collection of the Chinese Academy of Sciences (Shanghai, China) and cultured in RPMI 1640. The 293T cell line and TZM-bl cells, derived from HeLa cells with the HIV receptor CD4 and coreceptors CCR5/CXCR4, were maintained in Dulbecco’s Modified Eagle Medium (DMEM). All cell cultures were supplemented with 10% fetal bovine serum (FBS), 2 mM L-glutamine, 100 U/mL penicillin and 100 μg/mL streptomycin at 37 °C/5% CO_2_.

### Cytotoxicity determination *in vitro*

The effect of SFT and SAMT10 aqueous solutions on the viability of TZM-bl cells was assessed by monitoring MTT metabolism using a colorimetric assay for cell survival, which was performed using the method of Denizot and Lang.^21^ Briefly, cells were seeded at 10^4^/mL in 96-well flat-bottom microtiter plates with different concentrations of SAMT10 or SFT in medium solution. After continuous incubation for 48 h, 20 μL of MTT (5 mg/mL; equal to 10% of the culture medium volume) was added to the plate and incubated for 3 h at 37 °C. Then, the medium was removed, and formazan, a product generated by the activity of dehydrogenases in cells, was dissolved in solubilization solution [M-8910] equal to the original culture medium volume. The amount of MTT formazan was directly proportional to the number of living cells and was determined by measuring the optical density (OD) at 570 nm using a Bio Assay reader (BioRad, Hercules, CA, USA). Combination therapy was evaluated using serial dilutions of SAMT10 and SFT at a 1:2 ratio for the TZM-bl cell line. The ratio was determined by estimating the EC_50_ for each drug in initial experiments and by using these doses to determine the ratio for the combination therapy. Cell viability was calculated using the following equation: (OD treatment/OD control) × 100%. Experiments were performed in triplicate wells.

### Efficacy of SAMT10 and SFT *in vitro*

The antiviral activity of SFT and SAMT10 was determined against viruses pseudotyped with the envelope (Env) glycoprotein of the predominant HIV-1 strains currently circulating in China: HIV-1 subtype B, subtype C and CRF01_AE.^22^ Three HIV-1 Env-pseudotyped viruses were produced in 293T cells by cotransfection with the expression plasmid encoding Env of HIV-1 strains SVPB16 (subtype B), SVPC12 (subtype C) or SH1.81 (CRF01_AE), and the HIV-1 backbone plasmid expressing the entire HIV-1 genome except for Env, pNL4-3Δenv.^23, 24, 25^ The pseudoviral particles were collected, titrated and stored at −80 °C until use.

For the viral inhibition assay, ~1 × 10^4^ TZM-bl cells per well were plated in a 96-well plate in DMEM containing 10% fetal bovine serum and penicillin–streptomycin. After culturing at 37 °C overnight, 100 pseudoviruses at 50% tissue culture infective dose (TCID_50_)/well were mixed with either SFT or SAMT10 in PBS solution at the indicated concentrations. The mixtures were added to the cells in triplicate and then incubated at 37 °C in 5% CO_2_ for 48 h. The cells were lysed in the presence of Bright-Glo (Promega, Madison, WI, USA) and the relative luminescence was recorded using a Victor 3 luminometer (PerkinElmer, Waltham, MA, USA). Cells treated only with culture medium were used as a negative control. The EC_50_ for each single antiviral compound was then calculated using Compusyn software (Chou TC, NY, USA). The antiviral effect of SAMT10, SFT or the combination of both compounds on the HIV-1 wild-type strain IIIB (subtype B, X4; 100 TCID_50_/well) was investigated using MT4 cells, and the inhibitory effect was detected as described above.

### Supply and culture of human colorectal mucosal tissue explants

Mucosal tissue samples from the distal stump in colorectal cancer, after being confirmed negative via pathologic diagnosis, were collected from patients undergoing colectomy or rectectomy at the Shanghai Public Health Clinical Center (Shanghai, China). Written consent was obtained according to the Internal Review and Ethics Boards of the Shanghai Public Health and Clinical Center. The culture of human colorectal mucosal tissue explants was performed as previously described.^26, 27^ As described in a previous study,^28^ explants were pretreated with SAMT10, SFT or both for 20 min before exposure to HIV-1_IIIB_ (10^4^ TCID_50_/mL for colorectal mucosal tissue). The explants were incubated with the compounds for 2 h at 37 °C. The explants were then washed four times with PBS and transferred to fresh culture plates. After culturing without compounds overnight, the explants were again transferred to fresh culture plates and maintained for seven days with 50% medium feeds every two to three days. Free cells in the culture supernatant following the overnight culture were collected and washed twice with PBS before transfer to fresh plates and subsequent coculture with 4 × 10^4^ MT-4 cells/well to assess the ability of free cells to repress virus. Cultures were maintained for seven days, with 50% medium feeds every two to three days. Finally, HIV-1 infection was determined by assaying p24 in culture supernatants using an ELISA (HIV-1 P24 ELISA Kit, Hebei Medical University, Shijiazhuang, China).

### Statistics

EC_50_ values were calculated using Compusyn software (available for free download from www.combosyn.com). Inhibition data were analyzed for cooperative effects using the method of Chou and Talalay.^29, 30^ The equations and computer software used for data analysis were described previously.^29, 31^ The combination index (CI) equation, which accounts for both the potency (EC_50_ values) and shapes of the dose–effect curves (*m* values), is used to precisely analyze two-drug combinations. CI values are defined such that CI=1 indicates an additive effect and a CI<1 and a CI>1 indicate synergism and antagonism, respectively. Based on the actual experimental data, the software was used to calculate serial CI values over an entire range of effect levels (fraction affected, Fa) from 5% to 95%. These data were used to generate Fa–CI plots, which is an effect-oriented means of presenting synergism or antagonism.

Data were also analyzed using the isobologram technique, which is dose-oriented. The axes on an isobologram represent the doses of each drug. Two points on the *x*- and *y*-axes were selected to correspond to the doses of each drug necessary to generate a given Fa value. The straight line (hypotenuse) drawn between these two points on the *x*- and *y*-axes corresponds to the possible combination of doses required to generate the same Fa value, indicating that the interaction between the two drugs is strictly additive. If these drug combination points lie on a straight line, the effect is additive at that Fa value. If the point lies to the lower left of the hypotenuse, the effect is synergistic, and if the point lies to the upper right of the hypotenuse, the effect is antagonistic at that Fa value. The analysis was conducted in a stepwise manner by calculating the EC_50_ (or 75%, and 90% EC) values based on the dose–response curves of the single drugs (SAMT10 and SFT) tested separately or the two drugs tested in combination (SAMT10 and SFT). The CI was calculated using the median effect equation with the Compusyn software to assess the synergistic effect of the combinations. A CI of <1 indicates synergism, a CI of 1 or close to 1 indicates additive effects and a CI of >1 indicates antagonism.^27^

## RESULTS

### Cytotoxicity of SAMT10 and SFT

**Figure 2:**
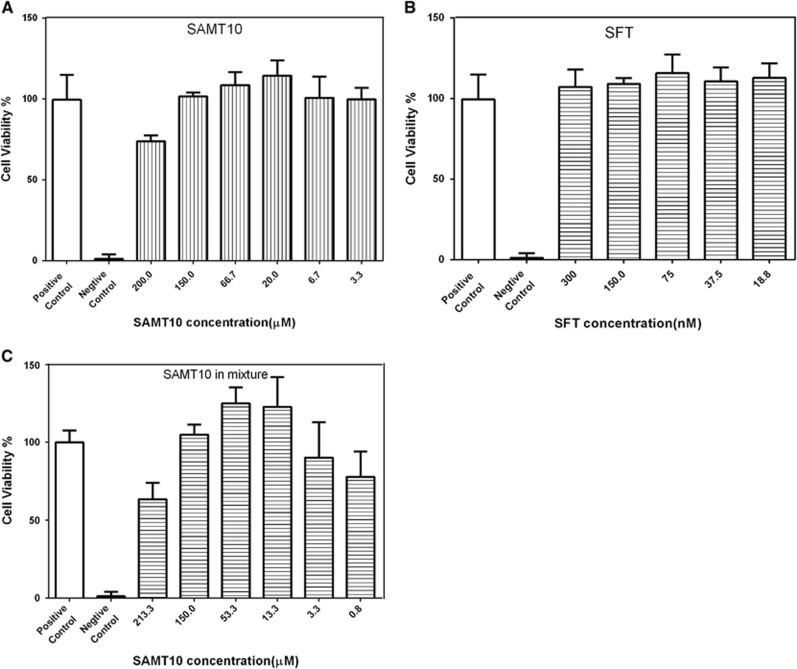
Assessment of SAMT10 and SFT cytotoxicity in cells. The viability of cells was tested for SAMT10 alone (**A**), SFT alone (**B**) or SAMT10 in combination with SFT (**C**). The range of doses for each compound tested was determined based on their EC_50_. Cell viability was quantified using the MTT method. Cells grown only in growth media served as the positive control, whereas cells cultured in 0.05% N-9 were the negative control. The percent cell viability was calculated by dividing the number of cells at each concentration of SAMT10 by the number of cells in the medium-only positive control. The results are representative of three independent experiments, and each experiment was performed in triplicate. The nontoxic dose of SAMT10 (maximal 150 μM) was used in subsequent experiments. 50% Effective concentrations, EC_50_; 3-(4,5-dimethyl-2-thiazyl)-2,5-diphenyl-2H-tetrazolium bromide, MTT; *S*-acyl-2-mercaptobenzamide thioester-10, SAMT10; sifuvirtide, SFT.

We evaluated the cytotoxicity of SAMT10 and SFT alone or in combination against TZM-bl cultured cells before testing its bioactivity to exclude the direct effects of compounds on the viability of TZM-bl cells. The cytotoxicity of SAMT10 was measured over a range of concentrations from 1 to 200 μM after 48 h of exposure. Compared with the negative control (medium only), SAMT10 at a concentration of 200 μM resulted in 73.84% viability (Figure 2A). Similarly, SFT at a concentration of 300 nM yielded 107.12% viability (Figure 2B). Furthermore, we observed 63.52% viable TZM-bl cells for SAMT10 tested at 213.3 μM combined with SFT (Figure 2C). Since anti-HIV bioactivity was measured at doses that were not cytotoxic (maximum 150 μM), we did not expect toxicity to confound the outcome of the antiviral activity assays.

### Combined use of SAMT10 and SFT shows greater potency than single inhibitor use in inhibiting HIV-1

Before analyzing the effect of the SAMT10 and SFT combinations, we first measured the antiviral activity of each compound alone on TZM-bl cells. For this, we used the following four viral strains: a laboratory-adapted HIV-1 strain (HIV-1_IIIB_ (subtype B, X4)) and three pseudotyped viruses (SVPB16 (subtype B, R5), SVPC12 (subtype C, R5) and SH1.81 (CRF01_AE, R5)). As shown in Table 1, SAMT10 exhibited EC_50_ values in the low micromolar range against all viral strains tested. This activity is consistent with the previous reports for this inhibitor in multiple cell types.^13, 28^ The antiviral activity for SFT was in the low nanomolar range, also consistent with the previous reports.^32^

**Figure 3:**
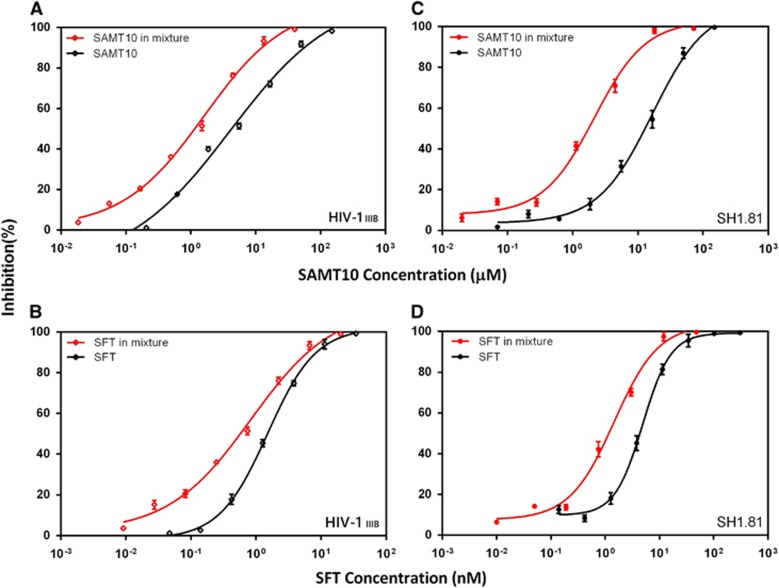
Efficacy of treatment of SAMT10 alone or in combination with SFT to prevent viral infection. (**A**, **B**) SAMT10 or SFT alone or in combination against the HIV-1_IIIB_ laboratory-adapted strain (subtype B, X4). (**C**, **D**) SAMT10 or SFT alone or in combination against HIV-1-pseudotyped virus SH1.81 (01_AE, R5). Plots of antiviral activity for SAMT10 or SFT alone are shown in black, whereas those of the combination are in red. These curves were fit to a sigmoidal curve using a nonlinear least squares regression to estimate the drug EC_50_. The range of doses for each compound tested was a 10-fold serial dilution based on EC_50_. The results are representative of three independent experiments, and each experiment was performed in triplicate. SH1.81 indicates the HIV-1-pseudotyped virus SH1.81. 50% effective concentration, EC_50_; *S*-acyl-2-mercaptobenzamide thioester-10, SAMT10; sifuvirtide, SFT.

We next examined the effect of combining SAMT10 and SFT to determine whether they have an additive or synergistic effect against infection by HIV-1_IIIB_ and the SH1.81 pseudovirus. The range of doses for each compound tested was 10-fold serial dilutions based on their EC_50_ values. Compared to SAMT10 alone, the EC_50_ value for the combination of SAMT10 and SFT decreased more than three-fold against HIV-1_IIIB_ and nearly six-fold against the SH1.81 strain (Figure 3, Table 2). This indicates that the inhibitory activity of SAMT10 combined with SFT was higher than that of SAMT10 alone against both strains. Compared to free SFT alone, the EC_50_ value for the combination of SFT decreased two-fold against HIV-1_IIIB_ and approximately five-fold against the SH1.81 strain. Similar to the results for SAMT10, the SFT results suggest that the inhibitory activity of the combined drugs is greater than that of SFT alone. Collectively, the dose reduction results indicate that SAMT10 and SFT used in combination show greater potency than either single-drug equivalent.

### Synergistic analysis of the combination of SAMT10 and SFT *in vitro*

**Figure 4:**
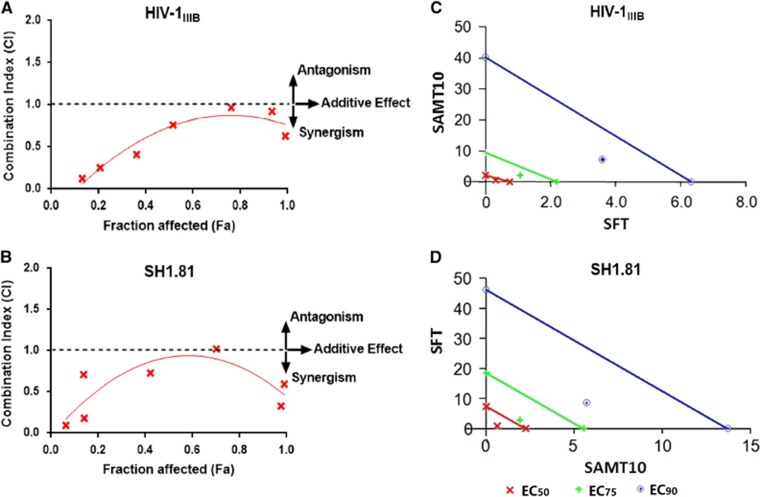
Combinatorial use of SAMT10 and SFT produces a synergistic effect for most doses evaluated. (**A**, **B**) The Chou–Talalay CI of the SAMT10 and SFT combination against the HIV-1 laboratory-adapted strain IIIB (subtype B, X4) and HIV-1 pseudovirus SH1.81 (01_AE, R5) was calculated. CI<1 indicates synergism. (**C**, **D**) Isobologram analysis of SAMT10 and SFT combination against the laboratory-adapted strain IIIB (subtype B, X4) and the pseudotyped virus SH1.81 (01_AE, R5), respectively. combination index, CI; 50% effective concentrations, EC_50_; 75% effective concentrations, EC_75_; 90% effective concentrations, EC_90_; *S*-acyl-2-mercaptobenzamide thioester-10, SAMT10; sifuvirtide, SFT. The results are representative of three independent experiments, where each experiment was performed in triplicate.

Two methods were used to assess the effect of SAMT10 and SFT combination, the CI method and the isobologram method. Using the Chou–Talalay method, we found that the CI values were <1 for four of the seven doses against HIV-1_IIIB_. For the SH1.81-pseudotyped virus, the CI values were <1 for all but the lowest dose (Figures 4A and 4B). This analysis indicates that moderate synergism was obtained against the HIV-1_IIIB_ strain, whereas strong synergism was observed against the SH1.81 strain. Isobolograms were further constructed for the doses of SAMT10 and SFT necessary to inhibit 90% of virus infection (EC_90_), as well as 75% (EC_75_) and 50% (EC_50_). The CI of each drug combination (Table 3) was plotted as a function of Fa using computer simulation from Fa=0.10 to 0.95. In this analysis, the combined effect at the 90% fractional inhibition (CI_90_) level was synergistic, additive or antagonistic when the CI<1, =1 or >1, respectively. When plotted, the experimental combination data points were at drug and viral concentrations well below the expected additive effect line for each of these Fa values (0.5, 0.75 and 0.9; Figures 4C and 4D). This second analytical approach confirms the synergistic effect of combining SAMT10 and SFT for both virus strains.

### Efficacy of the SAMT10 and SFT combination against HIV-1 infection in human colorectal mucosal tissues

The observed synergistic effects of combining SAMT10 and SFT *in vitro* led us to investigate the efficacy of SAMT10, SFT or the combination of both compounds on HIV-1 infection using human colorectal mucosal explants. Previous studies demonstrated that SAMTs could significantly block HIV-1 infection via either direct or trans pathways in vaginal explants,^28^ suggesting that combination with SFT may be efficacious in colorectal explants.

**Figure 5:**
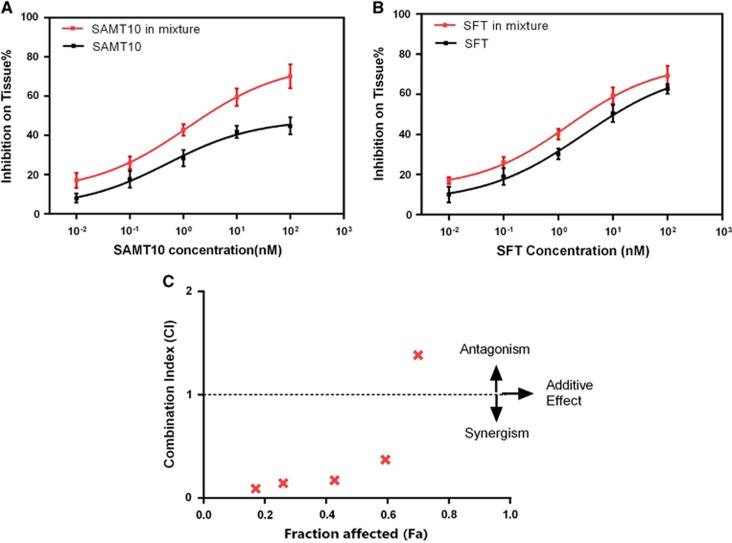
Combinatorial use of SAMT10 and SFT produces a synergistic effect in blocking HIV-1 transmission in human colorectal explants. The efficacy of treatment with SAMT10 or SFT alone compared with the combinatorial use of SAMT10 and SFT is shown in **A** and **B**, respectively. (**C**) Calculation of the CI value to assess the synergistic effect induced by the combinatorial use of SAMT10 and SFT in human colorectal mucosal explant models. CI<1.0 indicates synergism, CI=1 indicates an addictive effect and CI>1.0 indicates antagonism. The results are representative of three independent experiments, and each experiment was performed in triplicate. combination index, CI; *S*-acyl-2-mercaptobenzamide thioester-10, SAMT10; sifuvirtide, SFT.

When used alone, SAMT10 and SFT showed comparable activity in colorectal explants against HIV-1_IIIB_, as observed using cultured cells (Table 4). Similar to the findings in cultured cells, SAMT10 used in combination with SFT showed improved inhibition in human colorectal mucosal explant tissue compared to that of either inhibitor alone (Figures 5A and 5B). From the dose–effect curve, the combined activity of SAMT10 with SFT demonstrated a significant dose reduction. Compared to the treatment with SAMT10 or SFT alone, the EC_50_ values of the inhibitor combinations were reduced 1.5- to 2-fold in colorectal mucosal explants (Table 4). Analysis of the CI values plotted against the fractional effect of combination points using the Compusyn software indicated that the synergistic effect of this combination strategy increased the fractional inhibition at all but the highest dose (Figure 5C).

**Figure 6:**
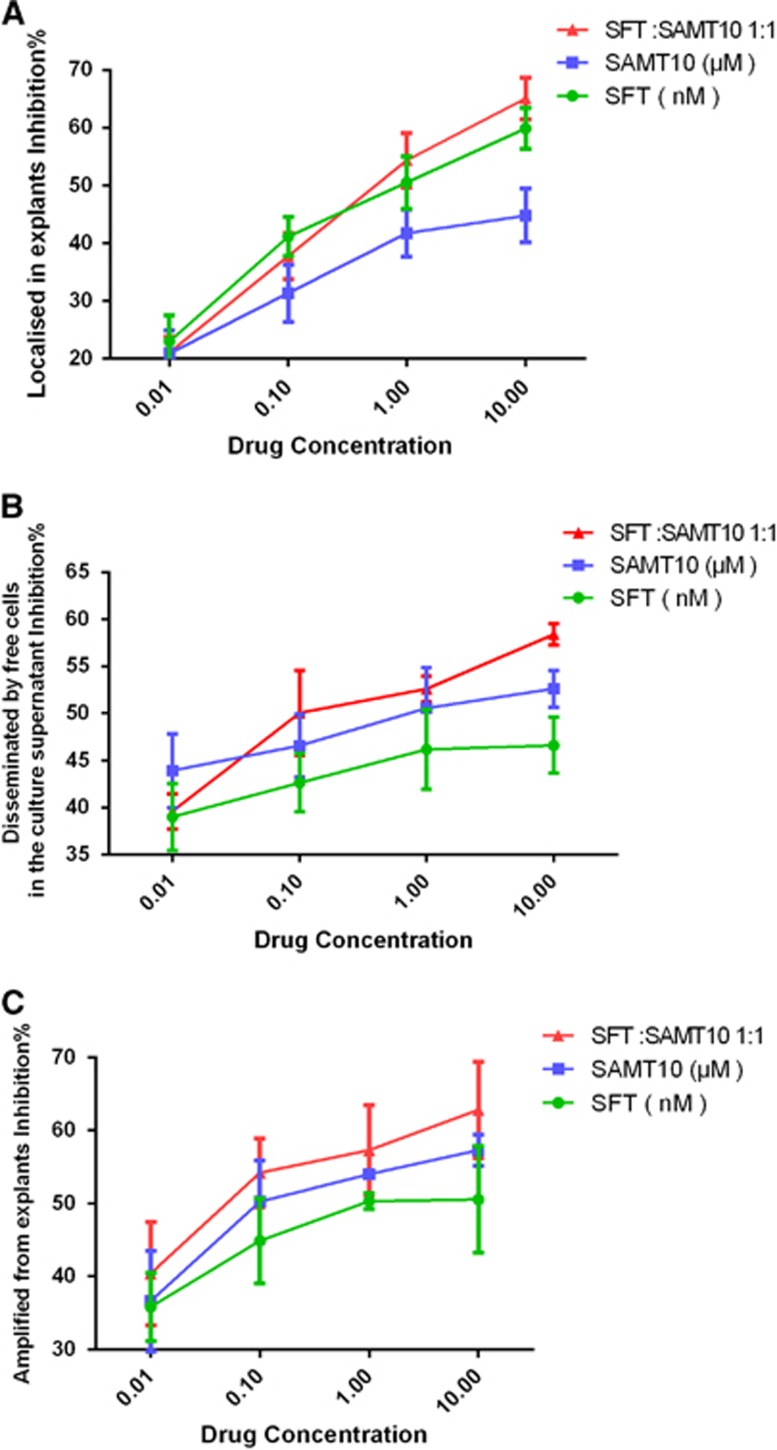
Effect of SAMT10, SFT or a combination of both inhibitors on HIV-1 infection in human colorectal mucosal explants. (**A**) The p24 concentration of the culture supernatant from colorectal mucosal explants with all free cells removed. (**B**) The free cells in the culture supernatant were washed and cocultured with MT4 cells in the absence of the compound. Explants were separated from any cells that migrated out of the explants and were cocultured with MT4 cells in the absence of compound (**C**). Measurement of p24 protein secreted into the supernatant on day 10 of culture using an enzyme-linked immunosorbent assay. The results are representative of three independent experiments, and each experiment was performed in triplicate. *S*-acyl-2-mercaptobenzamide thioester-10, SAMT10; sifuvirtide, SFT.

Furthermore, we confirmed the role of SAMT10 and SFT in the prevention of HIV entry and progeny virus transmission *in vitro*. In the current study, we found that SAMT10 in combination with SFT showed a synergistic effect on both the primary virus and progeny virus. As shown in Figure 6A, SFT significantly blocked viral transmission into the mucosa. SAMT10 mainly functioned as a potent inhibitor for the prevention of transmission of progeny virus (Figures 6B and 6C). Treatment with SAMT10, SFT or both, dose-dependently reduced p24 production in the culture supernatant separated from colorectal mucosal explants and in cells trans-infected by either free cells in the culture supernatant from the explant or the explant itself (Figures 6A–6C). The combination of SAMT10 and SFT led to significantly increased blockade of HIV-1 transmission.

## DISCUSSION

Relative to single inhibitor agents, attacking multiple targets could help combat HIV-1 sequence diversity and minimize the transmission of resistant variants.^9^ Although SAMT10 is efficacious against several strains of HIV-1, because of the susceptibility to HIV-1 variation, it is critical to assess its activity against novel variants that are region-specific. Moreover, the development of novel combinatorial preventive strategies is likely to be crucial for the prevention of new infections. SFT, a representative of the third generation of fusion inhibitors, has an effect early in the HIV-1 life cycle. In this study, when administered alone, SFT or SAMT10 exerted effective antiviral activity against the HIV-1 laboratory-adapted strain IIIB which replication was evidenced in the intestinal lymphocytes.^33^ Against the SH1.81-pseudotyped virus, the SAMT10/SFT combination showed strong synergistic effects. Therefore, the combination of SAMT10 with SFT showed favorable properties *in vitro*.

A previous study demonstrated that SAMTs can prevent viral transmission from infected cells by causing the release of noninfectious virons in cervical explants and primary cells, with EC_50_ values <50 nM.^28^ Although SAMT10 was not specifically examined in this study, as a member of the class, it is anticipated that SAMT10 will show similar activity. Indeed, we observed that SAMT10 prevents the infection of colorectal explants and blocks viral transmission when cells in the explant became infected, albeit at a higher EC_50_. SFT also showed good activity in colorectal explants, consistent with the previous findings in nonhuman primates.^19^ Moreover, when used in combination, the antiviral activity increased relative to either compound alone.

The results presented here demonstrate that synergistic effects were observed when SAMT10, a nucleocapsid inhibitor, was combined with SFT, a fusion inhibitor, to prevent infection by both a laboratory-adapted strain of HIV and a pseudotyped strain. Whereas SFT exerts its effect during the entry phase, SAMTs have both virucidal activity and effects late in the viral lifecycle. Thus, this type of combination is clinically valuable because SFT would inhibit the primary infection of cells and SAMT10 would prevent the transmission of the infectious virus from cells that escaped from SFT. This combination may be particularly useful for a rectal microbicide, which must protect a large area and a high number of potential target cells from infection. Since multivariant transmission of HIV is more likely to occur in receptive anal intercourse than in vaginal intercourse,^34^ the broad activity of SFT and SAMT10 against multiple strains of HIV could confer additional benefits to this combination. Thus, the combined use of fusion inhibitors and nucleocapsid inhibitors paves the way for the development of new microbicide combinations as an HIV prevention strategy.

## Figures and Tables

**Table 1:** SAMT10 and SFT EC_50_ in inhibiting HIV-1_IIIB_ and different subtype pseudoviruses

**Strains**	**SAMT10 (μM) (95% confidence interval)**	**SFT(nM) (95% confidence interval)**
B16	6.46 (4.42, 9.44)	4.20 (2.91, 6.08)
C12	2.30 (1.40, 3.77)	0.29 (0.19, 0.46)
SH1.81	6.28 (3.45, 11.43)	2.97 (2.30, 3.84)
IIIB	2.20 (1.32, 3.66)	1.10 (0.67, 1.84)

Abbreviations: 50% effective concentration, EC_50_; *S*-acyl-2-mercaptobenzamide thioester-10, SAMT10; sifuvirtide, SFT.

**Table 2:** The EC_50_ value of SAMT10 and SFT alone or in mixture in inhibiting HIV-1

**Virus type**	**SAMT10 (μM)**		**SFT (nM)**	
	**Alone**	**Mixture**	**Alone**	**Mixture**
SH1.81	6.23	1.06	3.52	0.71
IIIB	2.2	0.64	0.75	0.32

Abbreviations: 50% effective concentration, EC_50_; *S*-acyl-2-mercaptobenzamide thioester-10, SAMT10; sifuvirtide, SFT.

**Table 3:** CI value at different effective levels

**Virus type**	**CI at different inhibition levels**			**Synergy**^a^
	**50%**	**75%**	**90%**	
SH1.81	0.37	0.29	0.23	+++
IIIB	0.71	0.72	0.75	+

Abbreviations: combination index, CI.

a Synergy was determined at the EC_90_ level.

**Table 4:** The EC_50_ value of SAMT10 and SFT alone or in mixture in inhibiting HIV-1 in explants

**Virus type**	**SAMT10 (μM)**		**SFT (nM)**	
	**Alone**	**Mixture**	**Alone**	**Mixture**
IIIB	0.8598	0.3677	2.065	1.266

Abbreviations: 50% effective concentration, EC_50_; *S*-acyl-2-mercaptobenzamide thioester-10, SAMT10; sifuvirtide, SFT.
